# Reactive hyperemia and baseline pulse amplitude among smelter workers exposed to fine and ultrafine particles

**DOI:** 10.1007/s00420-019-01491-8

**Published:** 2019-11-26

**Authors:** Merete Drevvatne Bugge, B. Ulvestad, B. Berlinger, L. Stockfelt, R. Olsen, D. G. Ellingsen

**Affiliations:** 1 National Institute of Occupational Health, Oslo, Norway; 2 Unit of Occupational and Environmental Medicine, Department of Public Health and Community Medicine, Institute of Medicine, Sahlgrenska Academy, University of Gothenburg and Sahlgrenska University Hospital, Gothenburg, Sweden

**Keywords:** Cardiovascular disease, CVD, Endothelial function, Endo-PAT2000^®^, Metal smelters, Ultrafine particles

## Abstract

**Objective:**

Ambient exposure to fine particles is associated with increased cardiovascular morbidity and mortality. Associations between occupational particulate matter (PM) exposure and cardiovascular disease have been studied less. The objective of this study was to examine associations between PM exposure and endothelial function among workers in Norwegian smelters.

**Methods:**

We examined endothelial function with Endo-PAT equipment after a working day (WD) and on a day off (DO) in 59 furnace workers recruited from three metal smelters in Norway. The difference in baseline pulse amplitude (BPA) and reactive hyperemia index (RHI) between the 2 days was analysed in relation to individual exposure to PM < 250 nm (PM_250_) or the respirable aerosol fraction of particles, and adjusted for relevant covariates.

**Results:**

The exposure to PM_250_ ranged from 0.004 to 5.7 mg/m^3^. The mean BPA was significantly higher on WD relative to DO (772 vs. 535, *p* = 0.001). This difference was associated with PM concentrations among participants ≥ 34 years, but not among the younger workers. Reactive hyperemia was significantly lower on workdays relative to days off (1.70 vs. 1.84, *p* = 0.05). This difference was observed only among participants above the age 34. No associations with PM exposure were observed.

**Conclusions:**

PM exposure was associated with higher BPA among participants older than 34 years. BPA reflects microvessel pulsatility. Our results may indicate an age-dependent cardiovascular susceptibility to PM exposure. Endothelial function measured by RHI was reduced on WD among participants 34 years and older, but we found no associations between PM exposure and RHI.

## Introduction

Norway has, due to plentiful access to hydroelectric power, an old tradition for metal production. In some of these metal-producing plants, high concentrations of particulate matter (PM) may occur in the work environment, even though hygienic measures have been implemented to a great extent. During the years, several studies have shown increased risk of lung cancer and other lung diseases among metal-producing workers in Norway (Hobbesland et al. 1999; Johnsen et al. 2010; Søyseth et al. 2007), whereas the evidence for increased incidence of cardiovascular diseases (CVD) is sparse (Hobbesland et al. 1997).

Ambient air pollution is associated with increased incidence and mortality of cardiovascular diseases. Time-series studies estimate that a 10 µg/m^3^ increase in mean 24-h ambient PM < 2.5 µm (PM_2.5_) concentration increases the relative risk of cardiovascular mortality by 0.4–1.0% (Brook et al. 2010). Given the much higher PM exposure in metal-producing facilities than in urban air, CVD would be expected to be prevalent in the metal smelters.

Three main mechanisms have been proposed for the cardiovascular effects induced by PM exposure: (a) induction of local inflammation in the alveoli, “spilling over” into the systemic circulation, (b) triggering of pulmonary sensory receptors, resulting in autonomic nervous alterations, and (c) translocation of particles across the alveolar membrane, with a possible direct effect of particles on the vascular endothelium (Miller et al. 2012). Increasing concerns have been directed at the ultrafine component of PM. These concerns relate both to the large surface area relative to the mass, with the possibility of transporting large amounts of reactive substances into the alveoli, and to the small size (< 100 nm), possibly enabling the particles to translocate across the alveolar membrane into the bloodstream (Miller et al. 2012).

Endothelium is the innermost cell layer of the vascular system. Endothelial cells have a key role in the interaction of the vessel wall with circulating elements in the blood, as well as regulating vessel tone and growth, vascular inflammatory reactions, hemostasis, and coagulation (Deanfield et al. 2007). Conversely, the earliest detectable sign of cardiovascular disease is endothelial dysfunction (Ellins and Halcox 2011).

Endothelium releases vasodilating agents and induces hyperemia as a response to reduced blood flow to the tissue (Deanfield et al. 2007). This reactive hyperemia can be measured as increased peripheral arterial tone in the fingertips after occlusion of the brachial artery for a few minutes (Hamburg and Benjamin 2009). The baseline pulse amplitude (BPA) reflects microvessel pulsatility, and has been shown to correlate with important CVD risk factors, such as advancing age and higher blood triglyceride concentrations (Hamburg et al. 2008; Schnabel et al. 2011).

The aim of this investigation was to study the effect of exposure to respirable and ultrafine particles on reactive hyperemia and baseline pulse amplitude in furnace workers. More specifically, the hypothesis was that PM exposure on a working day would reduce reactive hyperemia and increase BPA, relative to a day off. This study is part of a larger study investigating effects of PM exposure on the cardiovascular health of smelter workers in Norway.

## Materials and methods

### Study design

To study the effect of respirable and ultrafine particles on epithelial function, we aimed to examine altogether 60 furnace workers from three metal smelters in Norway with Endo-PAT. The Endo-PAT2000 device (Itamar Medical, Israel) is a simple, non-invasive, operator-independent method for assessing both BPA and reactive hyperemic peripheral arterial tone (Axtell et al. 2010). Each furnace worker was to be examined twice; after a full-day shift at work (WD), and on a day off (DO), thereby making each participant their own control. Exposure measurements were performed on the same WD as the health examination was carried out. No air samples were collected on the DO, assuming a zero exposure on DO.

Two manganese smelters (plant A) and one silicon metal smelter (plant B) were contacted and invited to participate in the study, which all plants accepted. All plants practiced a full-shift work schedule, with five teams working in permanent rotating shifts. The recruitment of participants was organized by the health and safety management of each plant. They asked four workers at each shift team if they would voluntarily participate at both time points during the study, giving 20 participants from each plant. If a worker refused, or was unable to participate in the examinations, another worker was asked to take his or her place in the study. The main functions of the furnace workers were to control the tapping process (tappers) and to operate the cranes (crane operators). Tappers were monitoring the tapping process, either close (3–5 m) to the tapping hole, or nearby in a pressurized cabin. Crane operators performed their tasks mainly after the end of the tapping process, and 5–10 m further away from the tapping hole. Each tapping/crane driving process is performed three-to-four times during the shift, and takes 45–75 min each time. When they were not working at the tap floor, both tappers and crane drivers were resting in a control area, not exposed to PM {Berlinger, 2015 #870}. At two of the smelters, the furnace workers had either a tapper or crane operator function during their shift, whereas at the third plant, all workers performed both tapping and operating the cranes.

Each participant carried air sampling equipment during the whole WD shift. Health examinations were performed immediately after the 8-h day shift, starting around 14:00. The participants were re-examined at about the same time of the day (around 12:00) on a DO, after at least 2-day off work.

At inclusion, 65 potential participants were registered. One subject was excluded from the study due to serious health problems discovered during the first testing. Three participants were excluded from the final analyses, because they had worked an extra, unscheduled, night shift on the night before the DO testing. In addition, two participants had sickness absence from their second testing. Thus, the study comprises 59 subjects.

### Clinical examinations and Endo-PAT procedure

Demographics, height, and weight were recorded once. Blood pressure was measured once, in a seated position, to determine the required blood pressure cuff inflation during the Endo-PAT examination. A blood sample was drawn from the right arm at both examinations, before the Endo-PAT examination.

An Endo-PAT fingertip probe was placed on the index finger of each hand, and a blood pressure cuff was applied to the left overarm. The participants rested comfortably, while a physician interviewed them about current and previous work history, medical conditions, and potential confounders.

After a 10 min rest, the Endo-PAT procedure was initiated with fingertip probe inflation and a baseline recording of pulse amplitude for 7 min. Then, the blood pressure cuff was inflated until occlusion of the brachial artery (200 mm Hg or 60 mm Hg above systolic blood pressure, whichever was highest). The Endo-PAT recording continued with total occlusion of the pulse of the left index finger. After exactly 5 min, the blood pressure cuff was deflated, and the recording continued for another 7 min.

The Endo-PAT software automatically calculates the Reactive Hyperemia Index (RHI), which is the post-to-pre occlusion Peripheral Arterial Tone (PAT) signal ratio in the occluded arm, relative to the ratio in the control arm, corrected for baseline vascular tone of the occluded arm (Axtell et al. 2010). After the automatic analysis of the results, all recordings were manually checked. In four cases, the automated analyses gave no valid results because of artefacts in the registration. A manual setting of the time points for occlusion gave a valid analysis in two of these cases, whereas in the two other cases, no valid results could be achieved. When the automated analyses gave an occlusion duration outside the range 4.5–5.5 min (in nine cases), the borders of occlusion were set manually to 5 min. After reset, the Endo–PAT software recalculated RHI. The software also calculated the mean PAT signal amplitude in the baseline region of interest (BPA), both for the occluded and the control arm. Before statistical analyses, BPA was calculated as the mean between the two arms.

### Blood sampling and analyses

Blood was collected from the cubital vein in 8.5 mL vacutainer tubes (BD Vacutainer, Belliver Industrial Estate, Plymouth UK) and separated by centrifugation at 1900*g* for 15 min. All samples were pipetted into 1.0 mL NUNC^®^ polypropylene cryotubes (Sigma-Aldrich, St. Louis, Missouri, US) and stored in a freezer (− 18 °C) at the respective plants up to 5 weeks until transported to the National Institute of Occupational Health, Oslo, Norway (STAMI), where the samples were stored at − 80 °C until analysis.

Sample preparation and analyses of serum (S-) nicotine, S-cotinine, and S-caffeine were performed at STAMI, as previously described (Bast-Pettersen et al. 2017; Ellingsen et al. 2006). Detection limits (DL) were 31, 1.9, and 2.1 µg/L, for S-nicotine, S-cotinine, and S-caffeine, respectively. Before statistical analyses, results below DL were replaced by a value representing DL/2.

### Exposure measurements

Full-shift (8 h) respirable aerosol samples were collected by personal sampling with 37-mm respirable cyclones (JS Holdings, Stevenage, UK), using in-house built PS103 model personal sampling pumps (STAMI), at an airflow rate of 2.2 L/min. Sioutas cascade impactors (SKC, Eighty Four, PA, USA), using Leland Legacy model high flow personal sampling pumps (SKC, Eighty Four, PA, USA) operated at an airflow rate of 9 L/min, were mounted in parallel with a second 37-mm respirable cyclone, for the collection of aerosol fractions. The Sioutas cascade impactor separates PM on the impactor stages from the top to the bottom in the following aerodynamic particle diameter ranges (in μm): 10–2.5, 2.5–1.0, 1.0–0.5, and 0.5–0.25. Particles below the 0.25 µm cut-point are collected on an after-filter. Further details on the aerosol sampling have been published (Berlinger et al. 2015).

The collected PM was determined gravimetrically by a six-place Sartorius Micro model MC5 balance (Sartorius AG, Göttingen, Germany) in a climate-controlled weighing room dedicated to low filter mass measurements, at relative humidity 40 ± 2% and temperature 20 ± 1°C. DL were below 0.01 mg for all kinds of substrates and filters used in the study (Berlinger et al. 2015).

The finest aerosol fraction measured in this study was PM_250_, and we chose to perform the analyses of associations between exposure concentrations and Endo-PAT results using this fraction as a surrogate for ultrafine particles. In addition, we performed the same analyses using the respirable aerosol fraction (PM_Resp_), as this is a standard aerosol fraction used in many occupational studies.

### Statistical analyses

Variables with skewed distribution were log-transformed. Mixed model linear regressions were performed to study the ratio between geometric mean (GM) RHI and GM BPA between WD and DO. A fixed-effects linear regression model (Gunasekara et al. 2014) was constructed, using the ΔRHI or ΔBPA as outcome variables, and the individual exposure to PM_250_ or PM_Resp_ as exposure variables. Stepwise multiple linear regression analysis (backward procedure) was used to identify covariates for inclusion in the mixed- and fixed-effects models. The level of significance for inclusion was set to 0.10 (two-tailed), and the following covariates were included: age (dichotomized by the median: 19–33 vs 34–64 years) ΔS-caffeine, and Δ(time since last meal). Because of missing data in the covariates, the number of participants in the final analyses was somewhat reduced. Due to differences in PM air concentrations and participant age between the manganese plants and the silicon metal plant, stratification by metal production (into Plant A and Plant B) was also considered relevant in the further analyses. The level of significance in the mixed- and fixed-effects analyses was set to 0.05 (two-tailed).

All data analyses were performed using STATA 15 (StataCorp LP, Texas, USA).

## Results

### Participants

Subjects in plant B were younger, and had a higher prevalence of never-smokers and snuff-users, than subjects in plant A (Table 1). Mean body mass index (BMI) was similar among the subjects in Plant A and Plant B. The serum concentrations of nicotine and cotinine were higher among subjects in plant B, especially on working days, reflecting the high prevalence of use of nicotine containing snuff. Serum caffeine concentrations were higher in plant A, especially on days off (Table 1).

**Table 1:** Background data and concentrations of particulate matter < 250 nm or respirable aerosol fraction (PM_250_ or PM_Resp_), S-nicotine and S-caffeine among 59 metal smelter furnace workers, of whom 37 worked in Plant A and 22 worked in Plant B

	All		Plant A		Plant B	
	Mean	Min–max	Mean	Min–max	Mean	Min–max
Male sex (%)^a^	92	–	92	–	91	–
Age (years)^b^	37	19–64	42	20–64	29	19–54
BMI (kg/m^2^)^b^	26	20–38	26	20–38	26	20–35
Systolic blood pressure (mmHg)^c^	132	105–159	131	105–152	135	106–159
Diastolic blood pressure (mmHg)^c^	80	62–103	82	62–103	75	65–90
Never-smoker (%)^a^	41	–	22	–	73	–
Current smoker (%)^a^	29	–	32	–	23	–
Current snuff user (%)^a^	36	–	30	–	45	–
Exposure						
PM_250_ (mg/m^3^)^c^	0.19	0.004–5.7	0.053	0.004–0.59	1.6	0.17–5.7
PM_Resp_ (mg/m^3^)^c^	0.58	0.042–27	0.18	0.042–0.57	4.1	1.0–27
Work in furnace hall (years)^b^	13	0–40	17	0–40	7	2–27
Day off						
S-nicotine (µg/L)^c^	21	< DL-103	21	< DL-85	22	< DL-103
S-caffeine (µg/L)^c^	800	3.2–10,400	1180	3.2–10,400	420	4.9–6980
Coffee or caffeinated drink use last 6 h (%)^a^	49	–	63	–	27	–
Strenuous physical activity last 2 h (%)^a^	9	–	12	–	5	–
Time since last meal (h)^b^	5	1–24	5	1–18	6	1–24
Working day						
S-nicotine (µg/L)^c^	24	< DL-121	21	< DL-85	31	< DL-121
S-caffeine (µg/L)^c^	1370	< DL-11,300	1390	< DL-11,300	1340	68-10,200
Coffee or caffeinated drink use last 6 h (%)^a^	74	–	77	–	68	–
Strenuous physical activity last 2 h (%)^a^	24	–	30	–	14	–
Time since last meal (h)^b^	4	1–18	4	1–18	5	1–16

*BMI* body mass index, *DL* detection limit, *S* serum

^a^Prevalence. Smoker and snuff user categories are not mutually exclusive

^b^Arithmetic mean (AM)

^c^Geometric mean (GM)

### Exposure

The GM concentration of PM_Resp_ was 0.58 mg/m^3^ (min–max 0.042–27) (Table 1). The GM concentration of PM_250_ was 0.19 mg/m^3^ (min–max 0.004–5.7). The concentrations were higher in plant B than in plant A, both with respect to PM_Resp_ (GM 4.1 vs. 0.18 mg/m^3^) and to PM_250_ (GM 1.6 vs. 0.05 mg/m^3^). The correlation between the two particle-size fractions was high: Pearson’s *r *= 0.61 in plant A and Pearson’s *r *= 0.77 in plant B. The subjects had worked a mean of 13 years in a furnace hall (min–max 0–40).

### Endo-PAT

The unadjusted GM BPA was significantly higher on WD than on DO when all participants were considered (796 vs 545, *p* < 0.01) (Table 2), and in the age group 34–64 years, adjusted for covariates (Table 3). The unadjusted GM RHI was non-significantly lower on WD than on DO when all participants were considered (1.70 vs 1.82) (Table 2), and significantly lower in the age group 34–64 years, adjusted for covariates (Table 3). No consistent patterns were observed related to plants.

**Table 2:** Endo-PAT results among 59 metal smelter furnace workers, with paired sample *t* test of the difference between workday (WD) and day off (DO)

	Mean	Min–max
DO		
Pulse (beats/min)^a^	65	43–91
Reactive hyperemia index—RHI^a^	1.82	1.08–3.01
LnRHI^b^	0.60	0.08–1.10
Baseline pulse amplitude (BPA) occluded arm^a^	539	69–1710
Baseline pulse amplitude control arm^a^	523	58–1634
Baseline pulse amplitude (mean of both arms)^a^	545	64–1612
WD		
Pulse (beats/minute)^a^	67*	42–102
Reactive hyperemia index—RHI^a^	1.70	0.93–3.47
LnRHI^b^	0.53	− 0.08 to 1.24
Baseline pulse amplitude (BPA) occluded arm^a^	788**	53–2552
Baseline pulse amplitude control arm^a^	796**	103–2422
Baseline pulse amplitude (mean of both arms)^a^	796**	78–2487

*p < 0.05 between WD and DO

**p < 0.01 between WD and DO

^a^Geometric mean (GM)

^b^Arithmetic mean (AM)

**Table 3:** Mixed model of the ratio between workday (WD) and day off (DO) GM RHI and GM BPA among 57 metal smelter furnace workers, adjusted for S-caffeine and time since last meal

	*N*	BPA						RHI					
		GM_WD_	GM_DO_	Ratio	95% CI		*p*	GM_DO_	GM_WD_	Ratio	95% CI		*p*
All	57	772	535	1.44	1.17	1.78	0.001	1.84	1.70	1.08	1.00	1.17	0.05
Plant A	36	820	637	1.29	0.98	1.68	0.07	1.83	1.64	1.12	1.02	1.23	0.02
Plant B	21	685	418	1.64	1.13	2.39	0.01	1.85	1.83	1.01	0.86	1.18	0.91
Age < 34	27	592	577	1.03	0.80	1.32	0.85	1.76	1.80	0.98	0.88	1.09	0.72
Age ≥ 34	30	993	499	1.99	1.47	2.69	0.000	1.94	1.61	1.21	1.09	1.34	0.001
Males	52	841	582	1.45	1.17	1.78	0.001	1.84	1.69	1.09	1.00	1.18	0.05

Due to missing covariate data at both study points, two subjects were excluded from the analyses

*RHI* reactive hyperemia index, *BPA* baseline pulse amplitude, *GM* geometric mean, *CI* confidence interval

**Fig. 1:**
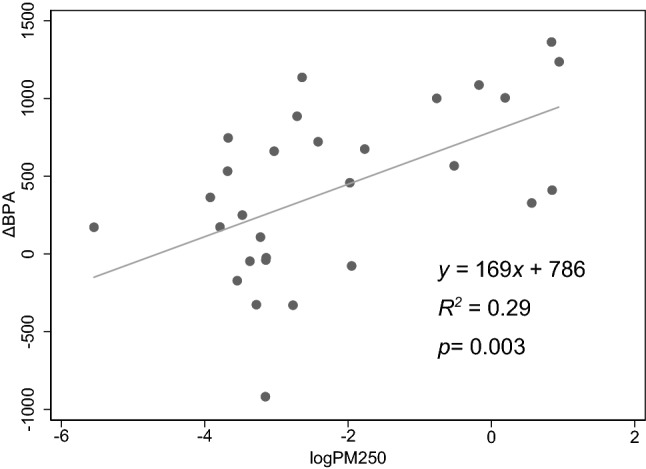
Association between mass exposure to particulate matter < 250 nm (logPM_250_) and difference in baseline pulse amplitude between workday and day off (ΔBPA) in metal smelter furnace workers aged 34–64 years

The results from the fixed-effects linear regression analysis of ΔBPA as dependent variable and exposure to either PM_Resp_ or PM_250_, and adjusted for relevant covariates as independent variables, are presented in Table 4. A statistically significant association between PM_Resp_ or PM_250_ air concentrations and increased BPA was observed among subjects in the age group 34–64 years (Fig. 1).

**Table 4:** Association between particle mass exposure (log PM_250_ and log PM_Resp_) and difference in baseline pulse amplitude (ΔBPA) and reactive hyperemia index (ΔRHI) between workday and day off among 48 metal smelter workers, using a fixed-effects linear regression model, adjusted for ΔS-caffeine and Δ(time since last meal)

	ΔBPA				ΔRHI				
	Coeff	95% CI		*p*		Coeff	95% CI		*p*
log PM_250_									
All	15	− 59	90	0.68	All	− 0.02	− 0.11	0.08	0.74
Plant A	− 17	− 252	218	0.88	Plant A	0.10	− 0.13	0.33	0.39
Plant B	77	− 256	410	0.63	Plant B	0.15	− 0.36	0.66	0.55
Age < 34	− 8	− 98	83	0.86	Age < 34	0.03	− 0.11	0.17	0.67
Age ≥ 34	152	43	260	0.009	Age ≥ 34	0.04	− 0.10	0.18	0.52
log PM_Resp_									
All	15	− 65	95	0.71	All	− 0.02	− 0.11	0.08	0.74
Plant A	− 65	− 350	219	0.64	Plant A	0.07	− 0.21	0.35	0.60
Plant B	61	− 238	360	0.67	Plant B	0.18	− 0.28	0.63	0.42
Age < 34	− 3.0	− 103	97	0.95	Age < 34	0.04	− 0.11	0.20	0.58
Age ≥ 34	178	62	294	0.004	Age ≥ 34	0.05	− 0.10	0.20	0.48

Due to missing covariate data, 11 subjects were excluded from the analyses

*CI* confidence interval, *PM(*_*250*_*or*_*Resp*_*)* particulate matter (< 250 nm or respirable aerosol fraction, respectively)

The results from the fixed-effects linear regression analysis of ΔRHI as dependent variable and exposure to PM_Resp_ or PM_250_, adjusted for relevant time-varying covariates, and stratified by relevant time-invariant covariates as independent variables, are presented in Table 4. No associations between PM exposure and reactive hyperemia were observed.

A large percentage of the RHI results, both on WD (57.6%) and on DO (42.6%), were below the level defined as “normal” according to the Endo-PAT manual. These “abnormal” results were evenly distributed between the plants. After end of occlusion, it took unusually long time (20–30 s) before pulse was registered in eight examinations. No associations were observed between extended missing pulse and relevant personal or exposure factors (not tabulated).

## Discussion

Few studies have been published on the associations between exposure to PM in the working environment and endothelial function. As such, this study represents an important contribution. As far as we know, this is the first study that aims to show associations between individual air concentrations of different particle-size fractions and RHI and BPA in metal smelter workers.

BPA was significantly higher after a WD relative to a DO. An association between PM air concentrations and ΔBPA was observed in subjects ≥ 34 years, also after adjustment for relevant time-varying covariates, but not among those < 34 years. RHI was significantly lower after a WD relative to a DO among the workers ≥ 34, but no associations with PM exposure were observed for ΔRHI.

### RHI vs BPA

Reactive hyperemia measured by Endo-PAT has for some years been considered a reliable indicator of endothelial dysfunction, which is an important risk factor for cardiovascular disease (Ellins and Halcox 2011; Hamburg and Benjamin 2009; Hamburg et al. 2008). It has, however, been suggested that BPA may represent a more relevant indicator than RHI of effects from PM exposure on the cardiovascular system (Hamburg et al. 2008; Ljungman et al. 2014; Schnabel et al. 2011). BPA reflects microvessel pulsatility, which is influenced by blood flow, autonomic tone, and vascular wall compliance (Ljungman et al. 2016). Positive associations have previously been observed between BPA and known CVD risk factors such as male sex, systolic blood pressure, BMI, body weight, fasting glucose, total/high-density lipoprotein (HDL) cholesterol, and current and previous smoking, but so far, associations between BPA and CVD have not been demonstrated in longitudinal studies (Sigurdardottir et al. 2017). Ljungman et al. found that higher short-term exposure to air pollutants, including particle number, PM_2.5_, and black carbon, was associated with higher BPA, suggesting that air pollutants may induce altered small-vessel tone or increase peripheral blood flow (Ljungman et al. 2014). Our results of increased BPA on WD relative to DO and significant associations with PM exposure among workers above the age of 34 are in accordance with that study.

Endothelial dysfunction in coronary and peripheral vessels is an independent predictor of cardiovascular disease (Lerman and Zeiher 2005). RHI assessed by Endo-PAT has shown high validity when compared to invasive “gold standard” methods (Bonetti et al. 2004). Inverse associations have been observed between RHI and important risk factors for CVD, such as diabetes, obesity, smoking, and the ratio between total and HDL cholesterol (Hamburg et al. 2008). Associations with PM exposures have been somewhat inconclusive: Decreased RHI was associated with increased air pollution exposure in elderly (Bräuner et al. 2008a; Zhang et al. 2016), but not in younger people (Bräuner et al. 2008b; Li et al. 2015; Ljungman et al. 2014). Exposures to ambient air pollution mixtures showed associations with BPA, but not with hyperemic response to ischemia (Ljungman et al. 2016). No effect on RHI was observed among welders compared to unexposed controls (Li et al. 2015).

In line with this, we found that RHI was reduced on a WD relative to a DO among participants 34 years and older, but not among the younger (Table 3). This difference, however, was not associated with increasing PM exposure (Table 4).

It should be noted that many subjects, also younger and apparently healthy workers, had an abnormally low RHI, both on WD and on DO. Similar levels of RHI have been reported also in other studies (Li et al. 2015; Ljungman et al. 2014). Explanations may be that field measurements at workplaces such as in this study or in Li et al. (2015) are, despite our best efforts, less well controlled than examinations in laboratory settings, that caffeine and nicotine were not eliminated, an unknown chronic pathology among the young workers, or perhaps a lack of relevant comparison data in the Endo-PAT software for this young, presumably healthy population.

### Covariates

Atherosclerosis progresses seamlessly from youth through middle age, and individuals who are susceptible to CVD have developed fibrous plaques in their arteries already by the age of 30 (McMahan et al. 2008). It is, therefore, probable that the cut-off at the median age 34 actually is biologically plausible. Both lifestyle and genetic factors may contribute to an increased CVD susceptibility (McMahan et al. 2008). It has also been suggested that exposure to ambient air pollution (Pope et al. 2004) and dust in occupational settings (Landen et al. 2011) may contribute to the development of atherosclerosis. In our study, we observed an association between PM air concentrations and ΔBPA among the workers with most years of previous furnace work experience (data not shown). The correlation between age and previous furnace work was, however, high, so the role of previous exposure cannot be evaluated in this study. The impact of a possible healthy worker effect should be considered. Our study shows an effect of exposure in the older part of our population. If there was a healthy worker effect making these older workers a selected population, this would mean that the effect of exposure is even greater than we observe. However, we are studying subclinical markers of CVD effects, where each participant is his/her own control. We have no reason to believe that there should be a healthy worker selection on the basis of subclinical cardiovascular effects.

Several other covariates were assessed in the study based on à priori knowledge. Ideally, the subjects should abstain from tobacco and coffee for some hours before the Endo-PAT examination (Neunteufl et al. 2002; Papamichael et al. 2005). However, this was a real-life study, and the results thus reflect the real-life situation. Therefore, information on coffee and caffeinated drink use last 2 h, smoking and snuff use, hours since last meal and tobacco use, and strenuous physical activity was collected and included as possible covariates in the analyses. In addition, serum caffeine, nicotine, and cotinine were determined. However, after elimination of covariates by backwards regression, only S-caffeine and time since last meal were observed to have a significant effect on the results.

Another covariate is sex, and BPA was much lower among females than among males (data not shown). The number of females participating in the study was too low to allow for stratification by sex in the final analyses. Exclusion of the females from the statistical analyses did not, however, noteworthy change the observed associations between exposure and outcome (Table 3).

Due to missing data in the covariates, the number of participants was reduced both in the mixed model and the fixed-effects regression analyses. Only 48 subjects had complete data from both examinations, which the fixed-effects regression analyses depend on. The mixed model can handle missing data, but two subjects were excluded also from the mixed model analysis, because one covariate was missing at both study points. We have no reason to believe that the missing data induced any bias in our analyses. However, the power of the study will suffer when the number of participants is reduced.

### Exposure measurements

This study has focused on the effect on endothelial function by exposure to respirable and ultrafine particles. For this purpose, exposure to different particle-size fractions was assessed by personal sampling with a five-stage cascade impactor (Berlinger et al. 2015). Ultrafine particles are defined to be smaller than 100 nm in aerodynamic diameter. Thus, our finest particle fraction contains more than only the ultrafines, but we consider the PM_250_-fraction a good surrogate for ultrafine PM exposure. We observed quite similar results when using PM_250_ or PM_Resp_ as markers of exposure, indicating that the effects of exposure were the same for the two particle-size fractions. However, the concentrations of the two particle-size fractions were highly correlated, and therefore, we cannot suggest any of the fractions as the main causal factor.

The chemical composition of PM exposure at the two plants is quite different, with both groups of employees exposed to a mixture of chemicals. At plant A, the submicron size fractions consist of MnO or MnSi, with smaller amounts of other oxides or silicates of Mn (Gjonnes et al. 2011). Particles from plant B are studied using electron microscopic (SEM) and atom spectrometric (ICP-OES) techniques, and they consist mainly of amorphous silica (Balazs Berlinger, personal communication). It is probable that the chemical composition of particles may influence their toxicological potential. However, we have, in this paper, chosen not to emphasise the chemical composition of the PM exposure. Our study hypothesis is based on previous observations of cardiovascular health outcomes among the general population exposed to particles in the outdoor air environment (Brook et al. 2010), where the chemical composition of particles is even more complex than in an industrial setting.

We have not measured the participants’ exposure to particles outside the workplace or on the day off, and we have made the assumption that this exposure is insignificant. The plants are situated in rural areas or in a small town, and workers do not live close to the plants, so we do not consider outdoor exposure as an important contribution to the total daily PM exposure of the employees.

Personal protection equipment will decrease the individual exposure relative to the aerosol measurements. The use of personal protection masks was mandatory in plant B, but not in plant A. Given that the aerosol concentrations were much higher in plant B than in plant A, and that personal protection masks do not protect completely from exposure (Janssen et al. 2007), the individual effective exposure may not have been very different between the two plants. However, by performing a stratification by metal production, we intended to bypass the possible bias induced by mask use.

## Conclusion

BPA was higher on WD relative to DO, and significant associations were observed with PM exposure among the participants 34 years and older, suggesting an effect of PM exposure on microvessel pulsatility among susceptible individuals. RHI was lower on WD among the subjects older than 34, but no association with PM exposure was observed.
